# Characterisation of Transcriptional Changes in the Spinal Cord of the Progressive Experimental Autoimmune Encephalomyelitis Biozzi ABH Mouse Model by RNA Sequencing

**DOI:** 10.1371/journal.pone.0157754

**Published:** 2016-06-29

**Authors:** Ioanna Sevastou, Gareth Pryce, David Baker, David L. Selwood

**Affiliations:** 1 Department of Medicinal Chemistry, UCL Wolfson Institute for Biomedical Science, London, WC1E 6BT, United Kingdom; 2 Neuroimmmunology Unit, Blizard Institute, Barts and the London School of Medicine and Dentistry, Queen Mary University of London, E1 2AT, United Kingdom; Westmead Institute for Medical Research, AUSTRALIA

## Abstract

Multiple sclerosis (MS) is a debilitating immune-mediated neurological disorder affecting young adults. MS is primarily relapsing-remitting, but neurodegeneration and disability accumulate from disease onset. The most commonly used mouse MS models exhibit a monophasic immune response with fast accumulation of neurological damage that does not allow the study of progressive neurodegeneration. The chronic relapsing and secondary progressive EAE (pEAE) Biozzi ABH mouse model of MS exhibits a reproducible relapsing-remitting disease course that slowly accumulates permanent neurological deficit and develops a post-relapsing progressive disease that permits the study of demyelination and neurodegeneration. RNA sequencing (RNAseq) was used to explore global gene expression in the pEAE Biozzi ABH mouse. Spinal cord tissue RNA from pEAE Biozzi ABH mice and healthy age-matched controls was sequenced. 2,072 genes were differentially expressed (q<0.05) from which 1,397 were significantly upregulated and 675 were significantly downregulated. This hypothesis-free investigation characterised the genomic changes that describe the pEAE mouse model. The differentially expressed genes revealed a persistent immunoreactant phenotype, combined with downregulation of the cholesterol biosynthesis superpathway and the LXR/RXR activation pathway. Genes differentially expressed include the myelination genes Slc17a7, Ugt8A and Opalin, the neuroprotective genes Sprr1A, Osm and Wisp2, as well as genes identified as MS risk factors, including RGs14 and Scap2. Novel genes with unestablished roles in EAE or MS were also identified. The identification of differentially expressed novel genes and genes involved in MS pathology, opens the door to their functional study in the pEAE mouse model which recapitulates some of the important clinical features of progressive MS.

## Introduction

Multiple sclerosis (MS) is a central nervous system disease that primarily affects young adults. MS has a median survival expectancy of 40 years after diagnosis [1], while patients start accumulating significant levels of disability early in disease progression, which adds a substantial burden to their everyday lives. MS is an immune-mediated disorder, characterised by demyelination and neurodegeneration [2]. The clinical presentation of MS is most commonly relapsing-remitting, but can also be primary progressive, where neurodegeneration and disability accumulate from disease onset. Over time relapsing remitting MS acquires a secondary progressive phenotype, with pathology accumulating between relapses [3]. Neurological symptoms in MS reflect focal inflammatory demyelinating lesions in the central nervous system that affect saltatory conduction in the affected sites [2]. Accumulating neurological disability is accompanied by disabling symptoms such as spasticity, tremor and muscle stiffness [4].

The most commonly used mouse model for the study of MS is the experimental autoimmune encephalomyelitis (EAE) model. This model has been extensively used in research, primarily in C57BL/6 mice immunised with myelin oligodendrocyte glycoprotein (MOG) peptide 35–55. These mice exhibit a monophasic immune response with fast accumulation of neurological damage, rendering them as perhaps not the most suitable EAE model to study progressive neurodegeneration [5]. The SJL mouse strain is a strain that develops relapsing-remitting disease following active immunisation, and is thus useful for the study of immune responses that more closely compare to the human disease [6]. However, the severe clinical disease and the low incidence of relapse in these mice makes it hard to study accumulating neurological disability. Thus existing mouse models do not always address the neurodegenerative processes that underlie primary or secondary progressive MS. The neurodegenerative component of MS remains a challenge and is currently untreated [7], in contrast to significant advances in controlling relapsing-remitting MS, which responds to immunosuppressive treatments [8–10]. Although the adaptive immune response drives relapsing disease and is sensitive to immune modifying drugs that act in the periphery to prevent new lesion formation and active attacks [7, 9], progressive neurodegenerative disease is probably driven by innate inflammatory responses within the CNS, which are insensitive to current peripheral immunomodulatory drugs in both progressive EAE and MS [7, 9, 11]. Additionally, it has been demonstrated that neurodegeneration can persist beyond the elimination of clinical relapses in EAE [12], as occurs in relapsing MS also [8]. Therefore there is a need to develop novel neuroprotective therapeutic approaches to complement current immunomodulatory approaches. To address this challenge, the study of chronic progressive EAE models, where the underlying pathological chronic neurodegenerative mechanisms are present, is expected to provide valuable insight in disease pathophysiology and potential therapeutic targets.

The chronic relapsing and secondary progressive EAE Biozzi ABH mouse model of MS exhibits a reproducible relapsing-remitting disease course that slowly accumulates permanent neurological deficit, which is followed by progressive neurodegeneration and disability (pEAE), with associated residual signs of disease such as spasticity and tremor [13–16]. Histological studies of Biozzi ABH mouse spinal cord tissue reveal immune-mediated relapsing disease episodes that prime the CNS-microenvironment for persistent demyelination, gliosis, glial cell activation, axonal and neuronal loss [16, 17]. Thus, the pEAE mouse model permits the study of mechanisms involved in the accumulation of neurological damage. Treatment of neurodegeneration is likely to be critical in the treatment of progressive MS so this Biozzi ABH mouse chronic model may provide a platform where strategies for neuroprotection and neurorepair can be evaluated.

In this study we used RNA sequencing (RNAseq) to explore gene expression in the spinal cord of the post-relapsing secondary progressive pEAE Biozzi ABH mouse model. During the post-relapsing pEAE disease stage the spinal cord is characterised by widespread demyelination, astrocytic gliosis, microglial activation and little to none immune infiltration [12, 17]. This hypothesis-free investigation of global gene expression aimed to characterise the genomic changes that describe this pEAE mouse model and to generate the complete transcriptome of the chronic, neurodegenerative EAE state. By studying the individual genes that were differentially expressed, as well as the pathways that were differentially regulated, we were able to better characterise the immunological, neurodegenerative and remyelinating components of the disease, as well as to propose genes and processes worth investigating further as potential therapeutic targets.

## Materials and Methods

### Animals

Adult (6–8 weeks), pathogen-free male Biozzi ABH mice were bred at Queen Mary University of London. Methods of housing and other reporting elements relevant to the ARRIVE guidelines have been reported previously [18]. All procedures were approved by the Queen Mary University of London Animal Welfare and Ethical Review Body and the United Kingdom Government Home Office Inspectorate. These studies where performed under Licence from the UK Home Office and conformed to the United Kingdom Animals (Scientific Procedures) Act 1986 for the use of animals in research and Directive 2010/63/EU.

### pEAE Induction

pEAE was induced in Biozzi ABH mice as described previously [18]. Briefly, young adult mice were injected subcutaneously with 1 mg freeze-dried mouse spinal cord homogenate in Freund’s adjuvant on days 0 and 7. Animals developed relapsing-remitting episodes of limb paralysis with remission. Spasticity and slow deterioration of movement typically developed after 2–3 relapses, ∼80–100 d post-induction [7, 9, 18]. Pathology in this model is largely restricted to the spinal cord [13], so animal spinal cords were sampled during remission from active paralytic episodes associated with hindlimb paralysis and weight loss [12, 17].

### RNA Extraction and Sequencing

Three post-relapsing chronic pEAE animals were sacrificed following the development of spasticity and tremor, at least 3 months after disease induction and after at least 3 clinical attacks as indicated previously [19]. Three age-matched control mice that did not receive an injection with spinal cord homogenate in Freunds adjuvant were also sacrificed. The spinal cord tissue was removed, snap-frozen in liquid nitrogen and stored at -80°C. Frozen tissue was disrupted in TRIzol^®^ Reagent on ice, using a rotor-stator homogeniser. Following 5 min incubation at room temperature, chloroform was added to the samples, which were shaken, left to rest and then centrifuged at 12000 g for 15 minutes. The resulting upper aqueous phase was washed with 70% ethanol, mixed well and loaded on an RNeasy column. Thereafter the Qiagen RNeasy^®^ Mini Kit protocol was followed to extract and purify mRNA. mRNA integrity was assessed by microfluidic capillary electrophoresis using the Agilent 2100 Bioanalyzer. All samples had a 260/280 ratio > 1.8 with RNA integrity number (RIN) > 9. RNA samples were processed and sequenced at the UCL Genomics facility (UCL Institute of Child Health) using the Illumina NextSeq 500 platform. Library preparation was performed using the TruSeq Stranded Total RNA Library Prep Kit. 43 bp paired-end sequencing was performed and approximately 18–25 million reads were obtained per sample. The dataset can be found in the Gene Expression Omnibus (GEO), accession number GSE78996.

## Data Analysis

The FASTQ files generated for each sample were aligned to the UCSC *Mus musculus* mm10 reference genome using the TopHat2 software (Illumina). Downstream analysis of these alignments was performed using Cufflinks software (Illumina). Cufflinks computes normalised fragments per kilobase of exon per million fragments mapped (FPKM) which reflect the expression levels of each mRNA molecule [20]. The reads were mapped to a total of 23,352 genes and 30,608 transcripts. To calculate *p* values, Cufflinks uses the Cuffdiff 2 algorithm that estimates expression at transcript-level resolution and controls for variability across replicate libraries. The Cuffdiff 2 statistical algorithm is described in depth in [21]. The Cuffdiff 2 algorithm statistically analysed gene expression and produced a list of differentially expressed genes in the pEAE tissue samples. The statistical analysis resulted in *p* values corrected for multiple testing with a default false discovery rate (FDR) of q <0.05.

To generate a heatmap for comparison of gene expression between all samples the UCL Genomics facility (UCL Institute of Child Health) R-based pipeline was used. To compare the lists of differentially expressed genes in pEAE with other published sets of genes, publically available Venn analysis software was used (http://bioinfogp.cnb.csic.es/tools/venny/index.html).

### Ingenuity Pathway Analysis

Ingenuity pathway analysis (IPA, Ingenuity Systems^®^, www.ingenuity.com) was used to identify biological and molecular networks differentially regulated in the pEAE model. IPA is a source of gene-interaction based pathway analysis including canonical pathways and a knowledge database based on scientific findings. Statistically significant differentially expressed genes at least 2-fold upregulated or downregulated were imported and analysed in the IPA database. Based on the direct or indirect connectivity of genes as disclosed in the literature, genes were mapped onto biological pathways and disease networks. Fisher’s exact test was performed to validate assignation of a biological function or disease to a network. Canonical pathway analysis was based on the identification of molecular pathways that are most significant for the dataset.

## Results

### Differential Gene Expression Levels

**Fig 1:**
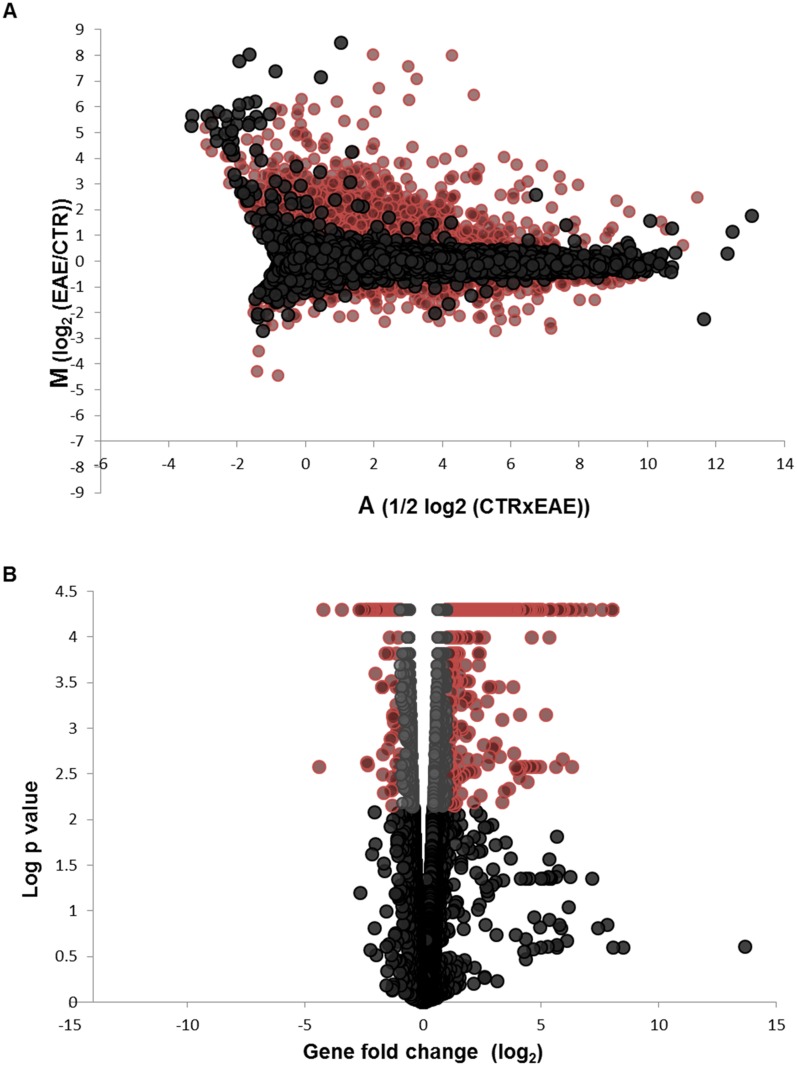
Identification of differentially expressed genes. (A) MA plot representing the ratio of FPKM expression values between chronic relapsing secondary progressive EAE samples and control samples plotted against their average. All 14,373 genes are plotted with significantly regulated genes (q<0.05) plotted in red. (B) Volcano plot presenting the 14,373 genes, with genes over the significance cut off at *p* <0.0072 (-log *p* <2.1426) plotted in grey. The statistically significant genes with >2-fold change in expression are plotted in red.

The spinal cord tissue RNA sequencing resulted in approximately 18–25 million reads per sample, which were aligned on the *Mus musculus* mm10 reference genome. This resulted in the identification of 23,352 annotated genes. Downstream statistical analysis of gene expression between control and pEAE samples (Cuffdiff) resulted in 14,373 genes successfully tested for differential expression (test status OK). Genes that were omitted from the analysis included genes with insufficient alignments for testing (NOTEST), too shallowly sequenced genes (LOWDATA), or genes with too many fragments aligned on the locus (HIDATA). It should be noted here that two genes, *Mbp* and *Plp1*, were not successfully tested due to a HIDATA return. These two genes are constituents of myelin, thus their involvement in this EAE model cannot be overlooked. They both had O FPKM in the control mice, but very high average fragment numbers (7939.56 and 2639.63 respectively) in the pEAE samples. Thus even though these genes are not included in the statistical analysis and the Ingenuity pathway analysis, they are included in the lists of highly upregulated genes. From the mapped genes, 2,072 were differentially expressed with a default false discovery rate (FDR) of q <0.05 (*p* <0.0072). More specifically 1,397 genes were significantly upregulated (q <0.05) and 675 genes were significantly downregulated (q <0.05) (S1 and S2 Tables). The differential gene expression in the pEAE samples compared with the control samples is visualised in Fig 1A. The MA plot presents the ratio of FPKM expression values between the two conditions. All 14,373 genes are presented in the plot with differentially regulated genes highlighted in colour. The volcano plot (Fig 1B) presents the 14,373 genes, with genes with FDR <0.05 (*p* <0.0072, -log *p* <2.1426) visualised in colour. In Fig 1B the statistically significant genes with a larger that 2-fold change in expression are presented in red and are the genes selected for further analysis. The heatmap in S1 Fig demonstrates the hierarchical clustering within the three control and three pEAE spinal cord samples which represents the differential expression of significantly regulated genes between the control and pEAE groups.

### Differential Gene Expression Analysis

The list of highly upregulated genes with a fold change of more than 16 (log_2_>4) reveals a number of genes with established roles in inflammatory processes, such as antigen processing and presentation, cell chemotaxis and cell adhesion (see S1 Table). This reveals a sustained inflammatory response in the spinal cord of the post-relapsing pEAE model, consistent with sustained microglial activity that remains following relapsing disease induced by the adaptive immune response [9, 12]. These findings are in line with the histological spinal cord studies during the progressive remitting stage of this EAE model, which have shown that the extensive immunoglobulin deposition and infiltration of macrophages, CD4^+^ T lymphocytes, B lymphocytes and leucocytes described during relapses is greatly diminished [13, 16] and the remitting spinal cord tissue is characterised by widespread demyelination, astrocytic gliosis and persistent low grade microglial activation [9, 12, 13, 17].

Additionally, a number of highly upregulated genes are genes involved in non-inflammatory biological functions, for example cell differentiation, proliferation, or ion transport. This list of genes is presented in Table 1. The Gene Ontology biological processes that are implicated in neurodegeneration, remyelination and related functions for each gene are also listed in Table 1. Some genes with cell adhesion properties may also be implicated in regenerative, remyelinating processes so they are included in the list. Associations with chronic EAE processes, differentiation, de/remyelination, neurodegeneration and neuroprotection are also referenced in Table 1.

**Table 1:** Most significantly upregulated genes (>16 fold change) with non-immunological functions.

Entrez Gene	Entrez Name	log_2_ fold change	*p* value	Gene ontology processes associated with EAE	Association with chronic EAE processes, differentiation, de/remyelination, neurodegeneration, neuroprotection
***Mbp***	myelin basic protein	inf	N/A	Myelination	Myelin constituent. Highly upregulated during oligodendrocyte differentiation [22].
***Plp1***	proteolipid protein (myelin) 1	inf	N/A	Myelination	Myelin constituent. Highly upregulated during oligodendrocyte differentiation [22].
***Mmp12***	matrix metallopeptidase 12	inf	5.00E-05	Positive regulation of epithelial cell proliferation involved in wound healing	Extracellular protease expressed in active macrophages in MS lesions [23]. Involved in the pathogenesis of Theiler’s murine encephalopathy, induces demyelination and neurotoxicity [24].
***Mcoln3***	mucolipin 3	inf	5.00E-05	Auditory receptor cell differentiation, ion transport	Transient receptor potential channel (TRPML3) involved in endocytosis [25], localized to lysosomes and initiates neutralised lysosome exocytosis [26].
***Atp6v0d2***	ATPase, H+ transporting, lysosomal V0 subunit D2	inf	5.00E-05	ATP hydrolysis coupled proton transport	Regulator of bone formation [27], no identified role.
***Gpnmb***	glycoprotein (transmembrane) nmb	7.09385	5.00E-05	Cell adhesion	Upregulated in Lewis rat EAE [28] and in an amyotrophic lateral sclerosis mouse model, proposed neuroprotective role [29].
***Wisp2***	WNT1 inducible signaling pathway protein 2	6.21785	5.00E-05	Cell adhesion, cell growth regulation	Promotes neurite outgrowth in ganglioside deficient mice [30].
***Plin4***	perilipin 4	5.82633	5.00E-05	Lipid storage regulation	Protein present in spinal cord, involved in lipid droplet storage [31]
***Steap4***	STEAP family member 4	5.37493	5.00E-05	Ion transport	Involved in osteoclast differentiation [32], no identified role.
***Ms4a7***	membrane-spanning 4-domains, subfamily A, member 7	5.34068	0.0001	Unknown	No identified role. Associated with late-onset Alzheimer’s disease [33]
***Gm4841***	*predicted gene 4841*	5.20993	0.0007	Unknown	No identified role.
***Cybb***	cytochrome b-245, beta polypeptide	5.02298	5.00E-05	Immune response, ion transport	Superoxide generating enzyme Nox2, implicated in microglial induced neurodegeneration [34]
***Gpr65***	G-protein coupled receptor 65	4.94688	0.0026	G-protein coupled receptor signaling pathway	Proton sensing TDAG8 receptor, involved in osteoclast regulation [35].
***Ch25h***	cholesterol 25-hydroxylase	4.91766	5.00E-05	Cholesterol metabolism	Proposed susceptibility gene for Alzheimer’s disease [36]. Proinflammatory enzyme, ch25h deletion attenuates EAE [37].
***Sprr1a***	small proline-rich protein 1A	4.60697	0.0026	Keratinocyte differentiation	Protein expressed after neuronal injury, involved in neuronal regeneration processes [38–40].
***Ppl***	periplakin	4.55594	0.0026	Unknown	Abundant in the brain, opioid receptor interacting protein [41], no identified role.
***Xdh***	xanthine dehydrogenase	4.4114	5.00E-05	Cell differentiation/apoptosis	Xanthine oxidase, the metabolic product of xanthine dehydrogenase, is Implicated in EAE pathogenesis, axonal and myelin loss [42].
***Wfdc17***	WAP four-disulfide core domain 17	4.38813	5.00E-05	Unknown	Anti-neuroinflammatory microglial protein [43].
***Osm***	oncostatin M	4.30594	0.0026	Immune response, peripheral nervous system development	Upregulated in relapsing-remitting MS patients [44], protective against demyelination in the Cuprizone-induced demyelination model [45].
***Ms4a6d***	membrane-spanning 4-domains, subfamily A, member 6D	4.26213	5.00E-05	Unknown	No identified role.
***Rnf186***	ring finger protein 186	4.0977	0.0007	Unknown	No identified role.
***Gpr114***	adhesion G protein-coupled receptor G5	4.09084	0.00345	G-protein coupled receptor signaling pathway	No identified role.
***Tnfsf11***	tumor necrosis factor (ligand) superfamily, member 11	4.07763	0.00275	Immune response, cell differentiation	Osteoprotegerin ligand RANKL, important osteoclast differentiation factor [46]. Upregulated in MS patients [47, 48].
***Serpina3i***	serine (or cysteine) peptidase inhibitor, clade A, member 3I	4.05241	5.00E-05	Unknown	No identified role.
***B430306N03Rik***	RIKEN cDNA B430306N03 gene	4.04922	5.00E-05	Unknown	Unknown protein.

The list of most significantly downregulated genes with a fold change of more than 4 (log_2_>2), reveals that no genes are directly involved in inflammatory processes. Most of the genes downregulated are involved in the cholesterol biosynthesis and metabolism superpathway. The list of the downregulated genes and their possible involvement in pEAE is presented in Table 2. The Gene Ontology biological processes that are implicated in neurodegeneration, remyelination and related functions for each gene are also listed, as well as associations with chronic EAE processes, differentiation, de/remyelination, neurodegeneration and neuroprotection.

**Table 2:** Most significantly downregulated genes (>4 fold change).

Entrez Gene	Entrez Name	log_2_ fold change	*p* value	Gene ontology processes associated with EAE	Association with chronic EAE processes, differentiation, de/remyelination, neurodegeneration, neuroprotection
***Ttr***	transthyretin	-4.43828	0.00265	Retinol metabolic process, thyroid hormone transport	Involvement in thyroxin transport, thyroxin promotes OPC differentiation [49], is protective in experimental autoimmune encephalomyelitis [50].
***Slc17a7***	solute carrier family 17 (sodium-dependent inorganic phosphate cotransporter), member 7	-4.25294	5.00E-05	Transport, synaptic transmission	Vesicle bound glutamate transporter (VGLUT1) localised in glutamatergic axon-OPC synapses and involved in myelination processes [51, 52].
***A930006I01Rik***	RIKEN cDNA A930006I01 gene	-3.47535	5.00E-05	Unknown	Unknown protein.
***Idi1***	isopentenyl-diphosphate delta isomerase	-2.70827	5.00E-05	Isoprenoid biosynthetic process	Cholesterol synthesizing enzyme, downregulated in Aβ treated neurons [53] and rat spinal cord injury [54].
***Hmgcs1***	3-hydroxy-3-methylglutaryl-Coenzyme A synthase 1	-2.61103	5.00E-05	Cholesterol metabolism	Cholesterol rate limiting enzyme, negatively regulated in rat spinal cord injury [54] and in a dysmyelinating mouse model [55]. Induces OPC migration and myelination failure [56].
***Fam216b***	family with sequence similarity 216, member B	-2.45922	5.00E-05	Unknown	No identified role.
***Ugt8a***	UDP galactosyltransferase 8A	-2.40356	5.00E-05	Myelination, lipid metabolism	Enzyme essential for myelination [57].
***Depdc1b***	DEP domain containing 1B	-2.403	5.00E-05	Cell migration	Promotes cellular de-adhesion and mitosis [58].
***9630013A20Rik***	RIKEN cDNA 9630013A20 gene	-2.34667	0.0025	Non-coding RNA	Unknown role.
***Mvd***	mevalonate (diphospho) decarboxylase	-2.30806	5.00E-05	Cholesterol metabolism	Enzyme involved in cholesterol metabolism.
***Sc4mol***	methylsterol monoxygenase 1	-2.25635	5.00E-05	Lipid metabolism	Enzyme involved in cholesterol metabolism and LXR signaling [59].
***Opalin***	oligodendrocytic myelin paranodal and inner loop protein	-2.23362	5.00E-05	Myelination	Myelin protein, located in paranodal loop membrane [60].
***Cyp51***	cytochrome P450, family 51	-2.17764	5.00E-05	Cholesterol metabolism	Enzyme involved in cholesterol metabolism.
***Slc5a11***	solute carrier family 5 (sodium/glucose cotransporter), member 11	-2.13978	5.00E-05	Ion transport	Glucose transporter implicated in immune modulation and apoptosis [61].
***Ldlr***	low density lipoprotein receptor	-2.13576	5.00E-05	Cholesterol metabolism	Involvement in LXR-induced myelination in oligodendrocytes [62].
***Aqp6***	aquaporin 6	-2.13396	5.00E-05	Transport	No identified role.
***Hmgcr***	3-hydroxy-3-methylglutaryl-Coenzyme A reductase	-2.12073	5.00E-05	Cholesterol metabolism	Rate controlling enzyme in cholesterol metabolism, upregulated in myelination [63], negatively regulated in a dysmyelinating mouse model [55].
***Sqle***	squalene epoxidase	-2.11207	5.00E-05	Cholesterol metabolism	Rate limiting enzyme in sterol biosynthesis.
***Smyd1***	SET and MYND domain containing 1	-2.10952	5.00E-05	Negative regulation of transcription	Key factor in myogenic differentiation [64], no identified role.
***4930452G13Rik***	RIKEN cDNA 4930452G13 gene	-2.03768	0.00025	Non-coding RNA	Unknown role.

### Gene Network Construction

**Fig 2:**
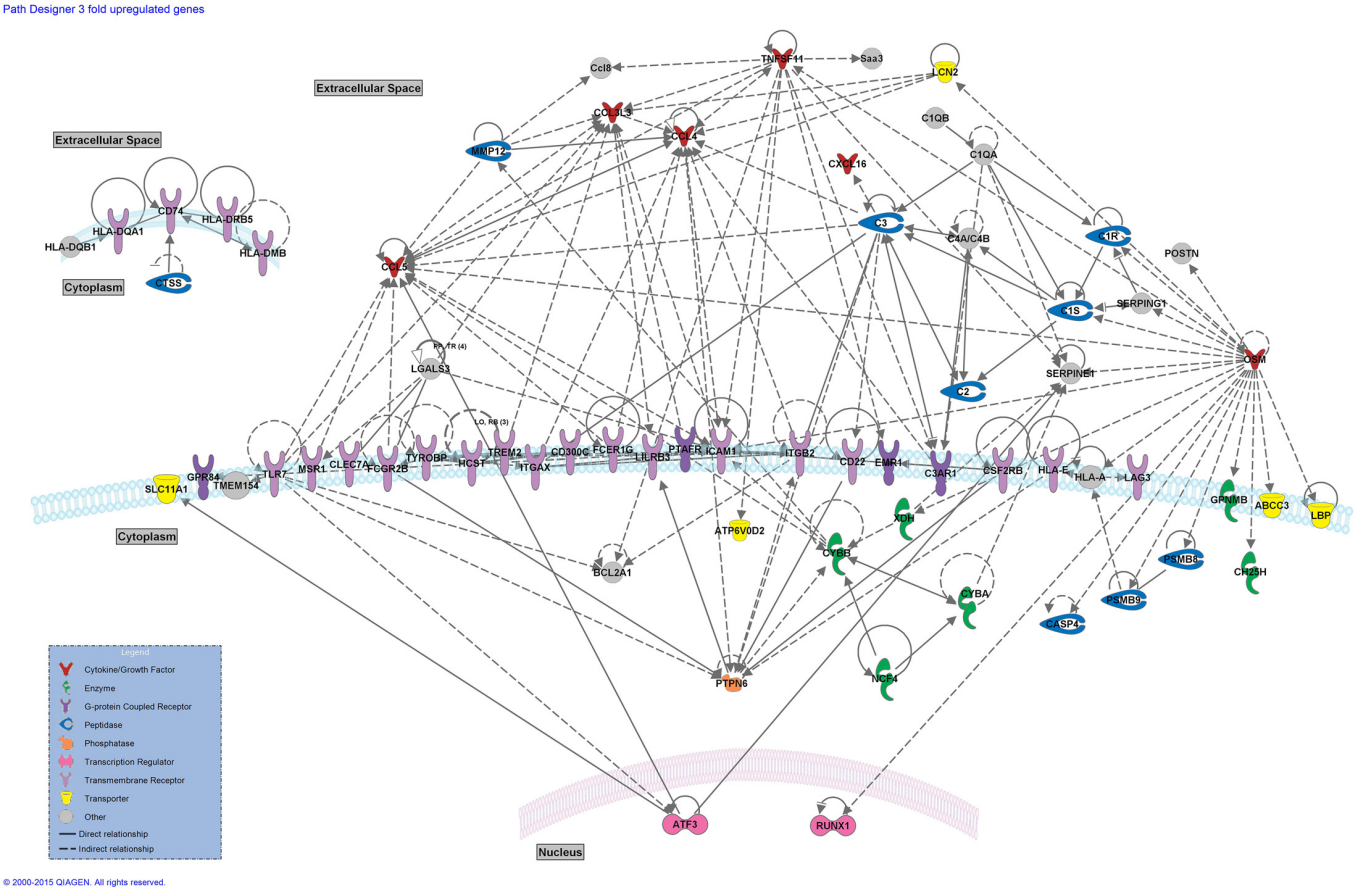
Network of direct and indirect gene connections between >3-fold upregulated genes. Network of direct and indirect gene connections as predicted by Ingenuity Pathway Analysis software. >3-fold upregulated mouse genes were uploaded on the IPA platform to establish a network of their published connections.

The gene network construction performed using the IPA platform aimed at creating a visual tool to assess connections between differentially expressed genes. The direct or indirect connectivity of genes as disclosed in the literature allows the assessment of connections between any two given genes. A network was constructed for the upregulated genes with >3-fold increase (Fig 2). A large number of functional direct and indirect connections can be seen between membrane receptors that take part in the response and maturation processes of the immune system. Membrane receptors that belong to the major histocompatibility complex (*H2* gene family receptors) group together since they directly interact with each other to orchestrate the autoimmune response. The constructed network illustrates the secreted chemokines *Ccl3l3*, *Ccl4* and *Ccl5* as hubs for multiple signaling connections. The connections between the upregulated members of the complement system, *C2*, *C3*, *C4a/C4b*, *C1s*, *C1r*, *C1qa* and *C1qb* are also depicted. Secreted *Tnfsf11* that encodes for the receptor activator of nuclear factor kappa-B ligand (*RANKL*) also acts as a hub for multiple gene connections. RANKL, encoded by Tnfsf11, and its receptor RANK are central in regulating the function of dendritic cells and are critically involved in the maintenance of the number and function of CD4^+^CD25^+^ regulatory T cells [65, 66] (also see discussion). The multiple indirect connections of the putative neuroprotectant oncostatin M (*Osm*; see discussion) are also presented. *Ptpn6*, a tyrosine phosphatase with a role in inflammatory disease also forms many direct and indirect connections with the upregulated gene population.

**Fig 3:**
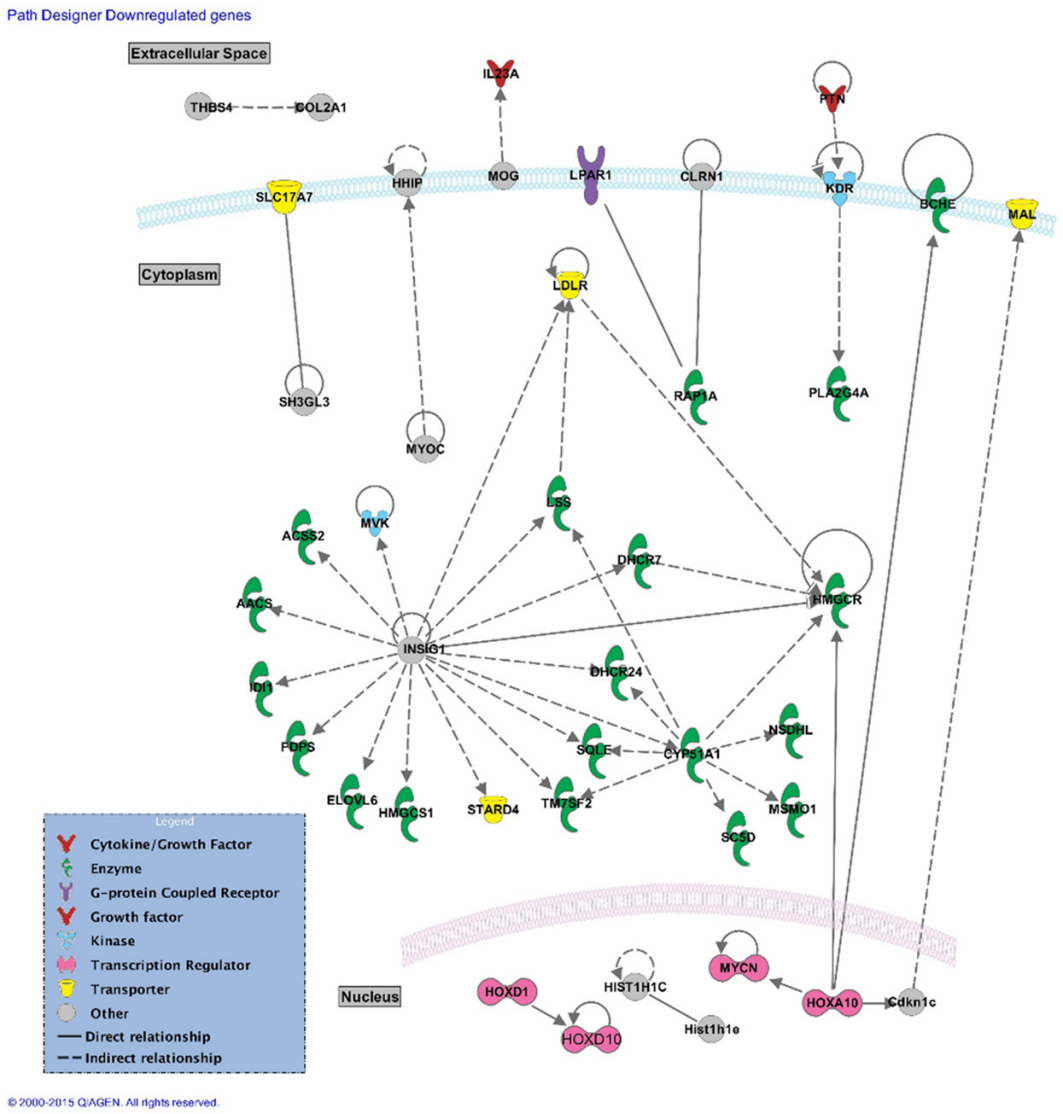
Network of direct and indirect gene connections between >2-fold downregulated genes. Network of direct and indirect gene connections as predicted by Ingenuity Pathway Analysis software. >2-fold downregulated mouse genes were uploaded on the IPA platform to establish a network of their published connections.

The number of significantly downregulated genes that were involved in direct or indirect connections was small, so this allowed for the construction of a network using all the significantly downregulated genes with >2-fold increase (Fig 3). Interestingly but not surprisingly, the gene that formed the most connections in the network was Insig1, an endoplasmic reticulum receptor that plays an important role in the downregulation of cholesterol biosynthesis. The connections between *Hmgcr* and *Cyp51a1*, both enzymes involved in cholesterol metabolism (see discussion), and other downregulated genes are also depicted in the constructed network.

### Canonical Pathway Analysis

For the canonical pathway analysis, differentially expressed genes with a FDR of q <0.05 and a fold change of at least 2 were selected (visualised in red in Fig 1B). This set of genes, comprising of 851 upregulated and 150 downregulated genes was imported into the Ingenuity pathway analysis platform (IPA, Ingenuity Systems^®^, www.ingenuity.com). The list of the 10 most significantly regulated canonical pathways is presented in Table 3. As expected from the gene analysis, the cholesterol biosynthesis superpathway was the most significantly downregulated pathway, with 17/27 genes downregulated. The LXR/RXR regulation pathway, a pathway emerging as a critical pathway in oligodendrocyte precursor cell (OPC) differentiation [67] was significantly regulated, with 23/121 genes upregulated and 4/121 genes downregulated. The rest of the significantly regulated canonical pathways were all upregulated pathways involved in immune processes, namely the dendritic cell maturation pathway, the T helper cell differentiation pathway, the pathway of hepatic fibrosis and hepatic stellate cell activation, the pathway of altered T and B cell signaling in rheumatoid arthritis, the acute phase response signaling pathway, the antigen presentation pathway, the complement system pathway and the pattern recognition receptors in recognition of bacteria and viruses. All the above pathways in many instances overlap, with upregulated genes involved in tumour necrosis factor signaling, toll receptor signaling or antigen presentation processes involved in more than one upregulated pathway. The upregulation of pathways involved in immune processes further supports the presence of persistently activated microglia in pEAE mice.

**Table 3:** Ten most significantly altered canonical pathways.

Ingenuity Canonical Pathways	-log(*p* value)	Ratio	Down-regulated	No change	Up-regulated	No overlap with dataset	Molecules
**Superpathway of Cholesterol Biosynthesis**	1.59E01	6.3E-01	17/27 (63%)	0/27 (0%)	0/27 (0%)	10/27 (37%)	HMGCS1, NSDHL, ACAT2, FDPS, HMGCR, SC5D, MSMO1, LSS, SQLE, IDI1, DHCR24, MVK, TM7SF2, HSD17B7, CYP51A1, MVD, DHCR7
**Dendritic Cell Maturation**	1.53E01	2.25E-01	2/169 (1%)	0/169 (0%)	36/169 (21%)	131/169 (78%)	PLCB2, H-2-AHLA-DQA1, FCGR1A, TNFRSF1A, PIK3R5, PLCE1, TLR9, FCER1G, NFKBIA, TREM2, TLR4, IL23A, IRF8, HLA-DMA, STAT1, TYROBP, HLA-DRB5, HLA-DMB, IL1RL2, CD80, TLR2, CD86, IL33, LTB, HLA-DQB1, TNFRSF1B, MAP3K14, HLA-DOB, B2M, FCGR2A, PIK3CG, PLCG2, HLA-A, FCGR2B, IL1A, COL2A1, ICAM1, IKBKE
**T Helper Cell Differentiation**	1.48E01	3.58E-01	0/67 (0%)	0/67 (0%)	24/67 (36%)	43/67 (64%)	IL21R, TGFB1, TNFRSF1A, HLA-DQA1, HLA-DQB1, TNFRSF1B, IL6R, ICOSLG/LOC102723996, HLA-DOB, FCER1G, IL2RG, IL10RA, TGFBR2, IL10RB, HLA-DMA, STAT1, HLA-DRB5, STAT6, HLA-DMB, CD80, IL4R, RORC, CD86, IL12RB1
**Hepatic Fibrosis / Hepatic Stellate Cell Activation**	1.31E01	1.94E-01	2/196 (1%)	0/196 (0%)	36/196 (18%)	158/196 (81%)	TGFB1, TNFRSF1A, IL6R, TNFSF8, LBP, TNFSF13, IL10RA, TLR4, A2M, CCR5, IL1R1, STAT1, EDN1, IL1RL2, LTB, CCL5, TNFSF11, COL6A3, KDR, TNFRSF1B, CD14, IGFBP5, FGF2, COL5A1, IGF1, IGF2, AGT, TGFBR2, COL23A1, CCL2, IL1A, IL4R, COL2A1, SERPINE1, FAS, COL13A1, ICAM1, HGF
**Altered T Cell and B Cell Signaling in Rheumatoid Arthritis**	1.27E01	2.96E-01	1/81 (1%)	0/81 (0%)	23/81 (28%)	57/81 (70%)	TLR7, TNFSF11, TGFB1, HLA-DQA1, HLA-DQB1, TLR1, MAP3K14, TLR9, FCER1G, TNFSF13, TLR4, IL23A, HLA-DMA, HLA-DRB5, IL1A, HLA-DMB, CD80, TLR6, FAS, TLR2, CD86, IL33, LTB
**Acute Phase Response Signaling**	1.16E01	1.96E-01	1/168 (1%)	0/168 (0%)	32/168 (19%)	135/168 (80%)	CFB, OSM, C3, SOCS3, TNFRSF1A, IL6R, LBP, C1R, NFKBIA, A2M, C4A/C4B, C2, IL1R1, VWF, ITIH2, IL33, CP, TNFRSF1B, RBP1, MAP3K14, SERPINA3, HMOX1, PIK3CG, OSMR, AGT, CEBPB, IL1A, FTL, SERPINE1, C1S, SERPING1, TTR, IKBKE
**Antigen Presentation Pathway**	1.16E01	4.32E-01	0/37 (0%)	0/37 (0%)	16/37 (43%)	21/37 (57%)	HLA-E, CD74, HLA-DQA1, NLRC5, HLA-G, HLA-DOB, B2M, TAP1, HLA-A, TAP2, HLA-DMA, HLA-DRB5, HLA-DMB, MR1, PSMB9, PSMB8
**LXR/RXR Activation**	1.1E01	2.23E-01	4/121 (3%)	0/121 (0%)	23/121 (19%)	94/121 (78%)	C3, TNFRSF1A, HMGCR, LBP, PON3, TLR4, C4A/C4B, LDLR, IL1R1, IL1RL2, IL33, MYLIP, S100A8, SREBF1, TNFRSF1B, CD14, APOD, ABCA1, MSR1, AGT, LYZ, CCL2, IL1A, LPL, CYP51A1, APOE, TTR
**Complement System**	1.06E01	4.17E-01	0/36 (0%)	0/36 (0%)	15/36 (42%)	21/36 (58%)	ITGAM, CFB, C3, C1QB, ITGAX, ITGB2, C1R, CFH, C3AR1, C1QA, C1QC, C4A/C4B, C2, C1S, SERPING1
**Role of Pattern Recognition Receptors in Recognition of Bacteria and Viruses**	1.04E01	2.18E-01	0/119 (0%)	0/119 (0%)	26/119 (22%)	93/119 (78%)	OSM, TGFB1, C3, TLR1, PIK3R5, TLR9, C3AR1, C1QA, TLR4, SYK, IRF7, TLR6, TLR2, PTX3, TLR7, CCL5, C1QB, CASP1, PIK3CG, PLCG2, C1QC, PRKCD, NLRP3, IFIH1, IL1A, CLEC7A

### Comparison between pEAE and Microarray EAE Studies

To dissect the unique molecular characteristics of the pEAE mouse model, its comparison with transcriptional studies of other EAE rodent models can provide valuable information about genes and pathways that may be similarly or differently regulated between different models. The comparison of the present pEAE study with other transcriptomic studies of EAE models proved difficult, since lack of publicly available data, especially from older studies, as well as differences in the methodology used, in animal species, strain and immunisation antigens selected, in most cases did not allow direct comparison of the studies. In absence of MOG-induced mouse EAE transcriptional data, the lists of differentially expressed genes from two EAE microarray studies re-analysed in a meta-analysis performed by Raddatz et al. [68] were selected to compare with the pEAE mouse data.

**Fig 4:**
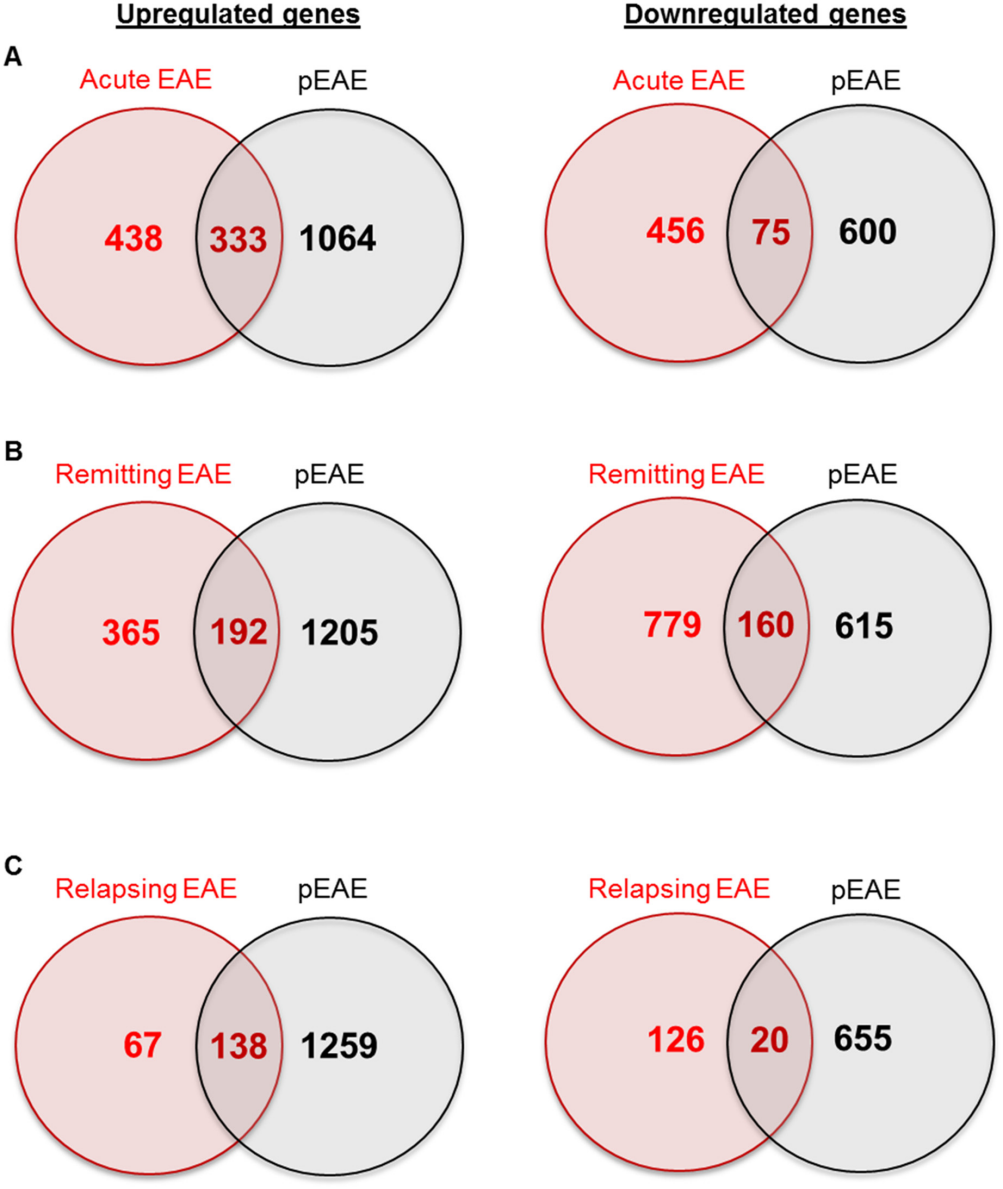
Comparison of the differentially expressed pEAE genes with differentially expressed genes in MOG-induced EAE in Dark Agouti rats at different disease stages. (A) Venn diagrams of upregulated and downregulated pEAE genes compared with acute MOG-induced EAE. (B) Venn diagrams of upregulated and downregulated pEAE genes compared with remitting MOG-induced EAE. (C) Venn diagrams of upregulated and downregulated pEAE genes compared with relapsing MOG-induced EAE.

The first study was a MOG-induced relapsing EAE study performed on Dark Agouti rat spinal cord tissue, where healthy rats were compared to the acute, remitting and relapsing states of the disease [69]. The comparison of the differentially expressed pEAE genes with the differentially upregulated or downregulated genes in the different disease states revealed some overlap between the two models (Fig 4). In the relapsing disease stage a large percentage of genes overlapped with the pEAE gene dataset. This supports higher similarities between the relapsing EAE phenotype and the progressive phenotype. All the lists of different or common genes at all disease stages can be found in S3 Table.

**Fig 5:**
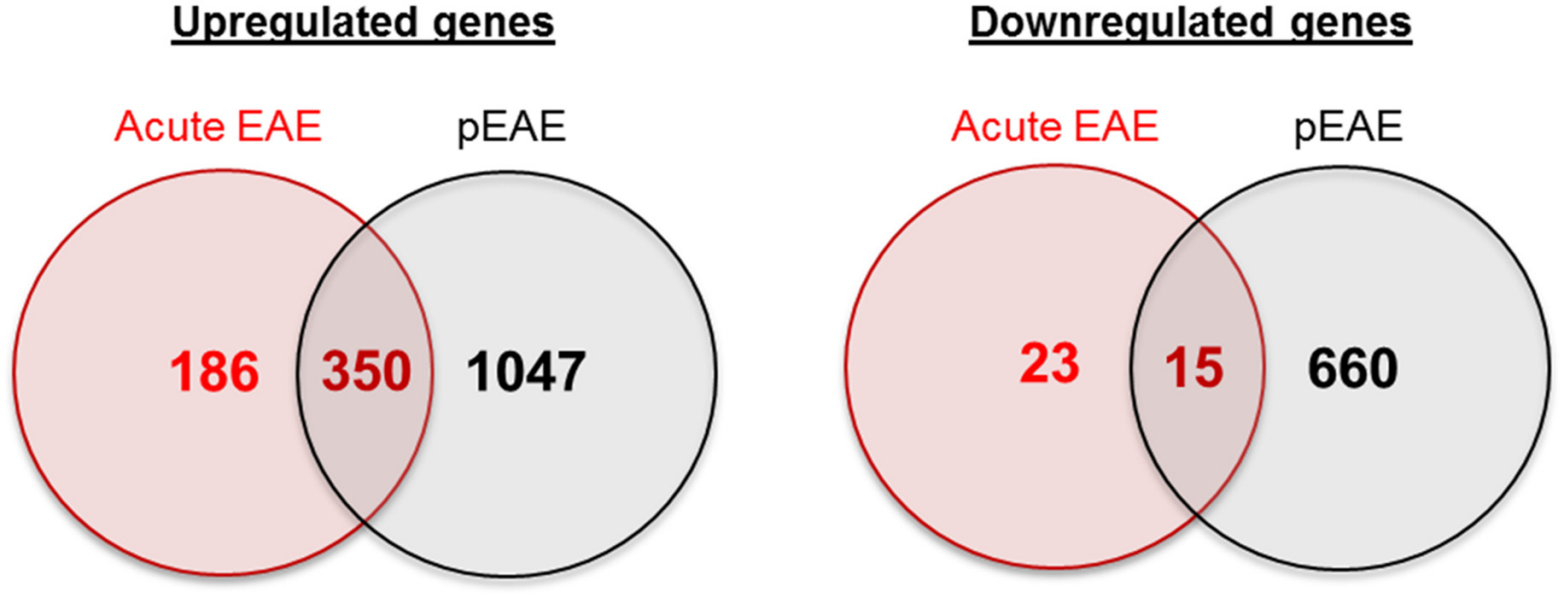
Comparison of the differentially expressed pEAE genes with differentially expressed genes in PLP-induced EAE in SJL/J mice during the acute disease phase. Venn diagrams of the comparisons between the upregulated and the downregulated gene datasets.

The second study was a PLP-induced acute EAE study of spinal cord tissue performed in SJL/J mice [70]. The comparison of the two different mouse EAE models revealed some similarities in gene expression (Fig 5). Since this comparison was performed between the acute disease stage of a monophasic EAE mouse model and the progressive EAE model, the lists of unique pEAE genes are informative in regards to the nature of genes differentially regulated in the chronic disease phase. The lists of different or common genes upregulated and downregulated in the two mouse EAE models can be found in S4 Table.

### Comparison between pEAE and Other RNAseq EAE Studies

**Fig 6:**
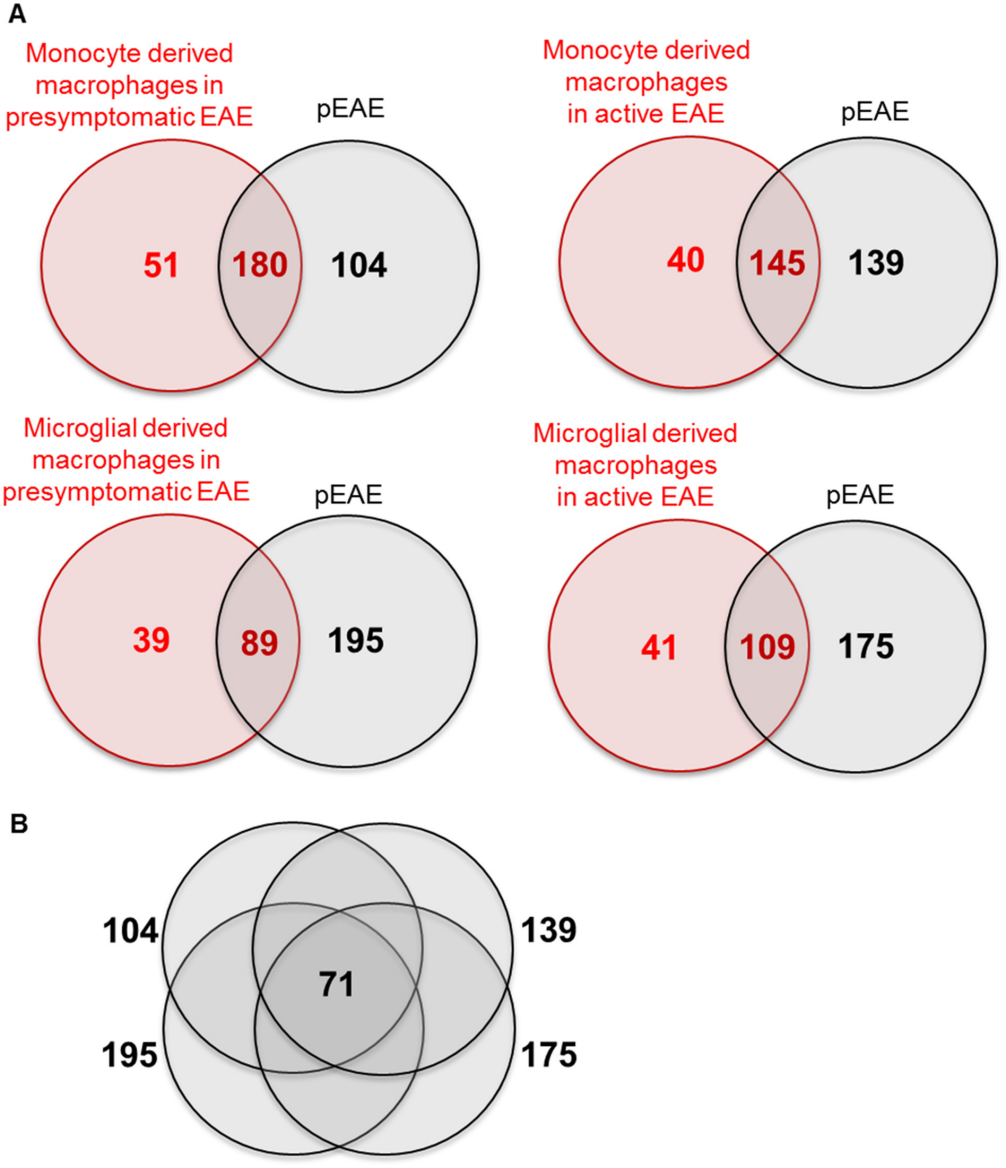
Comparison between the regulated pathways of the pEAE dataset with presymptomatic and active EAE (clinical score 3) monocyte derived macrophages, as well as with presymptomatic and active EAE microglial derived macrophages. (A) Venn diagrams of each comparison set. (B) Venn diagram of the unique canonical pathways regulated only in pEAE mice identified from the comparison of the chronic relapsing and secondary progressive EAE dataset with the macrophage populations.

RNAseq is a relatively new transcriptomic analysis technology and so far only very few RNAseq studies have addressed MS and EAE models. An advantage of RNAseq is that the very small amounts of RNA required allow the identification of the transcriptome of cellular populations rather than whole tissue. The single RNAseq study that investigated the whole transcriptome in an EAE mouse model was performed on spinal cord microglia and monocyte-derived macrophages isolated from different stages of MOG-induced EAE in C57BL/6 mice [71]. The differentially regulated canonical pathways identified in microglia and monocyte derived microglia during different disease stages were compared with our pEAE study. This comparison allowed us to identify the microglial and macrophage pathways that overlap with our dataset. It also allowed us to isolate pathways that are not enriched in microglial lineage cells and that can be related to monocyte/microglial independent disease mechanisms such as T-cell mediated immune processes, neurodegeneration, demyelination and remyelination. All canonical pathways in our dataset with 3 or more genes identified within the pathway were compared with the canonical pathways regulated in pre-symptomatic and active EAE (clinical score 3) monocyte derived macrophages, as well as with pre-symptomatic and active EAE microglial derived macrophages. As seen in the Venn diagrams in Fig 6A the majority of the canonical pathways enriched in each macrophage population were common with the pEAE dataset while some pathways were unique for the macrophage populations (all data in S5 Table). The unique canonical pathways in the pEAE dataset were plotted on a Venn diagram (Fig 6B) to isolate the 71 canonical pathways consistently unique to our dataset between all comparisons (S6 Table).

### Comparison between Differentially Regulated Genes and MS Susceptibility Genes

**Fig 7:**
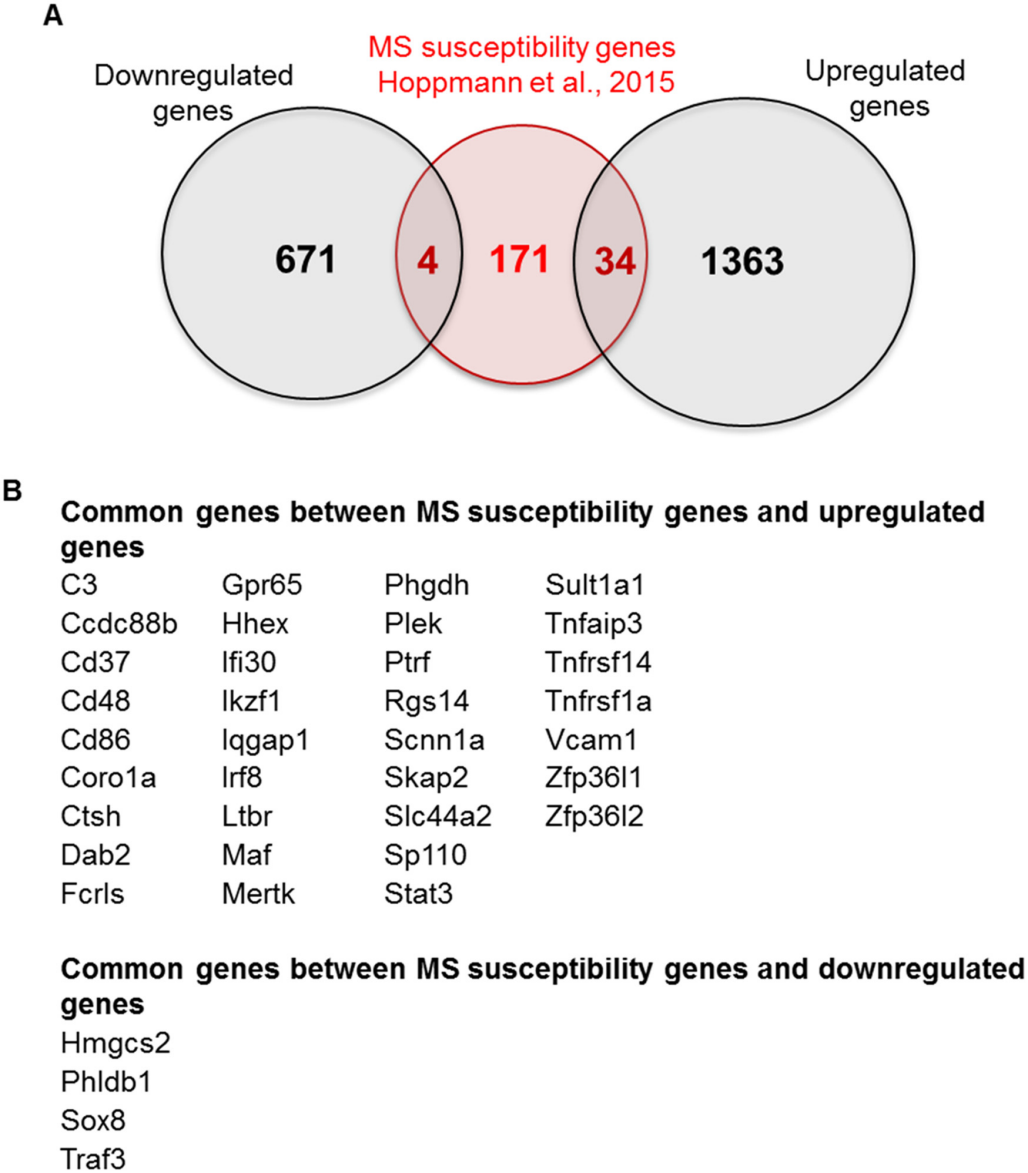
Comparison between the differentially upregulated and downregulated genes in the pEAE mice and MS susceptibility genes. (A) Venn diagram depicting the common genes between MS susceptibility genes (Hoppmann et al., [74]) and upregulated EAE genes (34) and the common genes between the MS susceptibility genes and downregulated EAE genes (4). (B) List of the common genes as identified in the Venn diagram.

During the last years, genome-wide association studies (GWAS) and other large-scale genotyping projects have revealed that only a few common genetic variants exist that exert relatively large MS risk, all of which are located in the *HLA* (human leucocyte antigen) locus. The remainder of the genetic risk spectrum comprises of a number of susceptibility variants exerting much smaller effects. So far, 110 independent SNPs outside the *HLA* locus have been identified to contribute to MS risk [72, 73]. A comparison between the published MS susceptibility genes and the differentially regulated genes in the pEAE mouse spinal cord tissue could reveal genes of specific interest to MS that also contribute to EAE pathology. Hoppmann et al. [74] recently produced a list of 209 human genes mapped in proximity to the 110 MS susceptibility loci. This list of mapped genes was compared with our set of upregulated and downregulated genes to identify MS susceptibility genes that overlap with our dataset. 34/209 MS susceptibility genes were significantly upregulated in the pEAE gene dataset, and 4/209 MS susceptibility genes were downregulated (Fig 7). These 38 MS susceptibility genes are of particular interest since their involvement in pEAE can highlight common disease processes.

## Discussion

The aim of this RNAseq study was to transcriptionally characterise the pEAE Biozzi ABH mouse model, to identify genes and molecular processes that may be specifically regulated in the chronic progressive phase of this disease model and to identify novel MS targets. The spinal cord transcriptional analysis revealed that a persistent immune response underlies the disease with immune pathways and genes highly upregulated (S1 and S2 Tables, Table 3). Immune pathways and responses established in EAE pathology are involved in the pEAE model, such as the dendritic cell maturation pathway, the T helper cell differentiation pathway, the antigen presentation pathway and the complement system. However, it is clear that the adaptive immune response driven by T cells that induces relapsing disease is reduced during progressive EAE, which is more associated with chronic microglial activation [9, 12, 17] as also appears to be the case in neurodegeneration in MS that is associated with chronic microglial activation [75]. Additionally, pathways and genes that seem to play an important role in the chronic disease progression were also revealed, such as the highly downregulated cholesterol biosynthesis and the RXR/LXR activation pathways. Genes that increase susceptibility to MS were found up- or downregulated in this model, providing insights into the relevance of this model for the study of MS processes.

### Genes Involved in Cellular Differentiation

Remyelination is a frequent event in MS lesions [76]. Shadow plaques, which are completely remyelinated lesions, account for 10–20% of all lesions [77]. Remyelination was histopathologically described in pEAE mice [17] and characterises the secondary progressive phenotype of this disease model. Here, the remarkable upregulation of *Mbp* and *Plp1* expression in the pEAE model confirms that active remyelination takes place during the progressive phase of the disease model. *Mbp* and *Plp1* encode for myelin basic protein and proteolipid protein 1, both constituent proteins of the myelin membrane, with a documented upregulation during oligodendrocyte differentiation [22]. Remyelination in progressive EAE as well as MS is limited. The reduced ability for lesion remyelination has been attributed to neuronal loss, but also the inability of OPCs to switch from a proliferating and migrating phenotype to a differentiating one. Genes involved in myelination significantly downregulated in the pEAE transcriptome reveal significant clues to remyelination failure processes. Vesicle bound glutamate transporter (VGLUT1), encoded by *Slc17a7* is an axon terminal glutamate transporter required in neuronal–OPC synapses to promote myelination processes [51, 52]. *Slc17a7* downregulation in pEAE reflects how neurodegeneration may inhibit myelination processes. *Ugt8a* was significantly downregulated in the pEAE mouse. *Ugt8a* encodes for the enzyme ceramide glucosyltransferase, which is essential for galactosylceramide production and myelin integrity [57]. Opalin is a myelin membrane protein present in paranodal loops [60] which was also significantly downregulated in the pEAE mouse. These dowregulated genes involved in myelination present as promising targets for new remyelination strategies.

Attention should also be drawn to some regulated genes involved in cellular differentiation processes with no identified role in EAE or MS. *Smyd1* for example is a downregulated transcriptional regulator identified as a key factor in myogenic differentiation [64] but with no known role in EAE or MS. Another example of a highly upregulated gene is *Mcoln3*, that encodes a transient Ca^2+^ channel, (TRPML3), which is involved in auditory receptor cell differentiation in mice [78]. Recently TRPML3 emerged as a transient receptor potential channel (TRP) located in lysosomes responsible for lysosomal extrusion following their neutralisation by bacterial infection [59]. The involvement of *Mcoln3* in lysosomal homeostasis could implicate it in autophagosomal processes that may be related to neurodegeneration. Another group of upregulated genes involved in cellular differentiation include the H^+^ transporting ATPase *Atp6v0d2*, the ion transporter *Steap4*, the proton sensing receptor *Gpr65* (TDAG8) and the Nf-κB ligand RANKL, encoded by *Tnfsf11* (tumour necrosis factor superfamily member 11), all involved in the regulation of osteoclast differentiation [27, 32, 35, 46]. The upregulation of osteoclast differentiation molecules may reflect defects in bone remodelling in pEAE and MS, or may reflect a yet unidentified involvement of this differentiation pathway in disease progression. It is interesting to note that RANKL is significantly upregulated in MS patient serum [47, 48]. RANKL and its receptor RANK have a critical role in regulating the function of dendritic cells and in maintaining the number and function of CD4^+^CD25^+^ regulatory T cells [65, 66]. The involvement of RANKL in T cell regulation in active EAE was demonstrated in a recent study where RANKL depletion prevented EAE development due to impaired T cell infiltration into the CNS [79]. Thus the upregulation of RANKL in our dataset and the upregulated protein levels in MS patient serum may reflect the involvement of RANKL in T cell regulation in EAE and MS.

### Genes Involved in Neurodegeneration and Neuroprotection

Some genes upregulated in the pEAE model that are involved in immune processes have been reported to also be involved in neurodegenerative processes. Matrix metallopeptidase 12 (*Mmp12*) is expressed in macrophages but has also been involved in inducing demyelination and neurodegeneration before macrophage infiltration in Theiler’s murine encephalopathy [24]. *Mmp12* was highly upregulated in pEAE highlighting the possibility that regulation of *Mmp12* levels could have a neuroprotective effect. Reactive oxygen species generating enzymes such as *Cybb*, encoding for the superoxide-generating microglial enzyme *Nox2* and xanthine dehydrogenase (*Xdh*) were also upregulated in pEAE. Both enzymes have been implicated in neurodegenerative processes [34, 42].

A gene with a well-documented role in neuroprotection was upregulated in the pEAE dataset. *Sprr1a*, the small proline-rich protein A1, is a protein involved in keratinocyte differentiation which is upregulated in neurons following experimental brain injury [38], and in sciatic nerve and spinal cord sensory neurons following axotomy [39]. *Sprr1a* promotes neuronal outgrowth and is expressed soon after neuronal injury. Thus the upregulation of this gene indicates the activation of a neuroprotective mechanism in the pEAE spinal cord and highlights a potential therapeutic avenue that deserves further investigation. The transient channel TRPML3, encoded by the *Mcoln3* gene and also known as cornifin-A, is a channel involved in keratinocyte differentiation. An additional gene involved in keratinocyte complex formation, Ppl which encodes periplakin was also upregulated in the pEAE mouse. It is possible that Ppl may be part of the pathway that promotes axonal regeneration together with Sprr1A, so further investigation of the involvement of *Plp* in neuroprotection would be of interest. Additional genes with neuroprotective roles were also upregulated in the pEAE mouse model. Oncostatin M, encoded by the *Osm* gene was found upregulated in the serum of a cohort of people with relapsing-remitting MS [44], while oncostatin treatment protected against demyelination in the Cuprizone-induced demyelination model [45]. *Gpnmb* is a transmembrane glycoprotein also found upregulated in Lewis rat EAE [28] and in a mouse model of amyotrophic lateral sclerosis [29]. A neuroprotective *in vitro* role has been proposed [29]. In a ganglioside deficient mouse model *Wisp2*, a protein with cell adhesion and cell growth regulation properties upregulated in our dataset was also found to be neuroprotective and promote neurite outgrowth *in vitro* [30]. The upregulation of neuroprotective genes in the pEAE mouse model suggests that this model can be used to investigate neuroprotective pathways in the progressive disease stage.

### The Cholesterol Biosynthesis Pathway

The cholesterol biosynthesis pathway was identified as the most significantly regulated canonical pathway in the Ingenuity pathway analysis (Table 3), with 17/27 genes assigned to this pathway significantly downregulated. Importantly, some of the cholesterol biosynthesis pathway genes, such as the genes encoding the cholesterol synthesising enzymes isopentenyl-diphosphate delta isomerase (*Idi1*), 3-hydroxy-3-methyglutaryl-coenzyme A synthase 1 (HMG-CoA reductase, Hmgcs1), methylsterol monoxygenase 1 (Sc4mol), 3-hydroxy-3-methylglutaryl-coenzyme A reductase (*Hmgcr*) and squalene epoxidase (*Sqle*) are in the list of the most significantly downregulated genes in this analysis (Table 2).

Significant downregulation of the cholesterol biosynthesis genes during relapsing disease has also been reported in MOG-induced EAE in Dark Agouti rats [69], as well as in the spinal cord of MBP-induced EAE in Lewis rats [28]. In contrast to the MOG-induced EAE study though, where only cholesterol biosynthesis genes were downregulated, in our model cholesterol transport genes were also downregulated, indicating downregulation of additional components of cholesterol metabolism in the pEAE model. HMG-CoA reductase, a cholesterol rate-limiting enzyme has also been found downregulated in people with MS [80]. In the dysmyelinating peroxisome-proliferator-activated receptor gamma coactivator 1 (PGC1α) knock-out mouse model, brain cholesterol, its precursors, and the rate-limiting enzymes HMG CoA synthase (*Mcoln3*) and HMG CoA reductase (*Hmgcr*), were downregulated [52]. There are two possible mechanisms that may result in cholesterol biosynthesis downregulation in EAE. One mechanism may involve the presence of cholesterol at lesions due to the breakdown of the cholesterol-rich myelin membranes, leading to inhibition of its biosynthesising pathway and contributing to remyelination inhibition. Cholesterol negatively regulates the transcription of cholesterol biosynthesis genes [81]. Another mechanism involved in cholesterol biosynthesis downregulation involves the activation of the immune system. It was recently shown that metabolic factors involved in cholesterol biosynthesis are downregulated to promote the expansion and reorganisation of pro-inflammatory CD4^+^ Th17 cells *in vivo* [74].

The involvement of cholesterol synthesis rate-limiting enzymes in MS and EAE pathology is also highlighted by the fact that HMG-CoA inhibitors ameliorate EAE and MS relapses by inhibiting immune cell activation and migration through the blood-brain barrier [82]. Lovastatin and simvastatin are HMG-CoA inhibitors taken to clinical studies for relapsing remitting MS [83, 84]. However, more importantly, high dose simvastatin has recently been shown to inhibit neurogeneration in secondary progressive MS [85], which is a disease stage that is not typically responsive to immune activation or blockage of peripheral immune responses entering the brain [8]. The finding here that cholesterol biosynthesis pathways are highly dysregulated in pEAE, which is mostly unresponsive to peripheral immunomodulation, may support the value of HMG-CoA inhibitors in progressive MS and provide a tool for mechanistic studies to understand the neuroprotective effects of statins. The transcriptomic data available clearly indicate a downregulation of this pathway but further investigation is needed to determine the physiological consequences of this downregulation.

Additionally *Ch25h*, a gene involved in cholesterol metabolism, was upregulated in the pEAE mouse. Cholesterol 25-hydroxylase is involved in lipid metabolism, catalyses the formation of 25-hydroxycholesterol from cholesterol and represses cholesterol biosynthetic enzymes [86]. Thus its upregulation agrees with the preferential downregulation of the cholesterol biosynthesis pathway. *Ch25h* has also been proposed as a susceptibility gene for Alzheimer’s disease [36] and its deletion can significantly attenuate EAE disease course by limiting trafficking of pathogenic CD4^+^ T lymphocytes to the central nervous system [37]. Lastly a lipid storage regulator, *Plin4* is upregulated in pEAE and with no identified role in EAE or MS pathology its potential role in cholesterol or LXR/RXR metabolism (see below) would be worth investigating.

### The LXR/RXR Activation Pathway

The LXR/RXR activation pathway was also significantly regulated in the pEAE model with 23/121 genes involved in this pathway downregulated and 4/121 upregulated. Genes involved in this pathway include *Ttr* (transthyretin), the most significantly downregulated gene in this study (Table 2), the HMG-CoA reductase gene, as well as the highly downregulated genes S*c4mol* and *Ldlr*. The liver X receptors (LXRs) and the retinoid X receptors (RXRs) are obligate heterodimers that form ligand-activated nuclear transcription factors that regulate lipid homeostasis, including cholesterol metabolism [87]. The LXR pathway is emerging as a critical pathway in oligodendrocyte precursor cell (OPC) differentiation [67]. It was found that the RXR-γ receptor was significantly upregulated in the regenerative phase of remyelination in a toxin-induced demyelination model in rats, while the LXR/RXR activation pathway was significantly regulated [67]. This positive regulation of remyelination is a promising pharmaceutical target. The suppression of the LXR/RXR activation pathway identified in the pEAE model, may contribute to the limited remyelination observed in this model and suggests that upregulation of relevant components of the LXR/RXR pathway may promote remyelination.

Interestingly and perhaps not surprisingly, the LXR/RXR activation pathway was also highly regulated in the transcriptomic analysis of T cells isolated from acute mouse EAE [74], implicating LXR/RXR signaling in immune cell responses. In addition to genes such as *Srebf1*, *Hmgcr*, or *Cyp51a1* which are involved in cholesterol biosynthesis, genes such as *Abca1*, *Srebf1* and *Lpl* which are involved in cholesterol transport were also found regulated in Th17 cells, as well as in our dataset, implicating cholesterol transport in the disease progression. mRNA levels of LXR-β were also found increased in MS patient peripheral blood mononuclear cells [88]. Additionally, combination of LXR and RXR or PPARγ and RXR agonists can inhibit microglial and astrocyte inflammatory responses *in vitro* and in EAE models [89–92]. LXR/RXR signaling provides promising therapeutic options in MS, but much is still unclear about the role of this signaling pathway in specific cell populations and different phases of disease progression. Transcriptomic analysis of acute and chronic models can help with the elucidation of LXR/RXR signaling in MS.

Transthyretin, encoded by the *Ttr* gene is associated with the LXR/RXR activation pathway and is highly downregulated in our dataset. Transthyretin transfers thyroxin from the blood to the brain, where thyroxin is essential for oligodendrocyte maturation [49]. Thus the low transcripts of transthyretin in EAE spinal cord may be one factor contributing to remyelination failure. Transthyretin is highly expressed in MS patient serum [93], suggesting that there may be a failure in transthyretin transfer from the blood to the CSF and the brain and that pharmacological enhancement of that pathway may increase thyroxin availability in the brain and spinal cord and promote remyelination in MS patients.

### The pEAE Mouse Model Transcriptome Compared to Other EAE Models

The pEAE Biozzi ABH mouse model exhibits a reproducible relapsing-remitting disease accompanied by demyelination, gliosis, glial cell activation, axonal and neuronal loss, followed by a slowly accumulating permanent neurological deficit [16, 17], which was examined here. The pEAE mouse model has been used for the study of mechanisms involved in the accumulation of neurological damage. Genes and pathways relevant to MS pathology are identified in this model, but its comparison to other EAE models can provide a lot of information about genes and processes unique to this model or common with other progressive phenotypes. The comparison of pEAE with a MOG-induced relapsing EAE study in Dark Agouti rats during the acute, remitting and relapsing stages of the disease [69] revealed some similarities between the two models, and especially similarities between upregulated genes (Fig 4, S3 Table). 66% of the upregulated genes in the relapsing phase of the MOG-EAE model were common with the pEAE model, opposed to 42% in the acute phase and 33% in the remitting phase. This supports higher similarities between the relapsing EAE phenotype and the pEAE phenotype, as such active inflammation and progressive lesions co-exist in progressive MS, although the equilibrium is shifted to chronic glial cell lesions [75]. A limitation of our study was that only tissue from the secondary progressive phase of the disease was studied. Additional studies of different disease stages of the pEAE mouse model would help identify the molecular pathways unique in the progressive stage, as well as give even more information regarding similarities and differences between relapsing-remitting EAE models. On the other hand, our study identified a larger number of differentially expressed genes in comparison to older microarray studies.

Another interesting subject for further investigation would be the identification of the transcriptome of the different cellular populations of the pEAE spinal cord. RNAseq analysis has made it possible to collect small amounts of RNA and perform transcriptomic analysis. The comparison of our study with an RNAseq study of the transcriptome of monocyte derived or resident CNS microglia [71] in MOG-induced EAE revealed a wealth of information about macrophage-specific pathways regulated in pEAE, as well as pathways uniquely identified in pEAE (Fig 6, S6 Table).

### MS Susceptibility Genes Significantly Regulated in Progressive EAE

pEAE is an experimental autoimmune mouse model that recapitulates key pathological features of secondary progressive MS, such as demyelination, remyelination, gliosis, axonal and neuronal loss [17]. Genes significantly modulated in pEAE are involved in disease mechanisms that are similar to MS pathology. To identify molecules in pEAE that may point to molecular pathways specifically relevant to MS pathology, we compared the pEAE transcriptome with 209 MS susceptibility genes [74] and identified 39 significantly regulated genes that are MS susceptibility genes (Fig 7). This list of genes (Fig 7B) comprises of immune-related genes such as the ligand *C3*, the cell surface proteins *Cd37*, *Cd48*, *Cd86*, *Fcrls*, *Ltbr*, *Vcam1*, *Ccdc88b* and the cytoplasmic proteins *Coro1a*, *Ifi30*, *Plek*, *MertK and Dab2*. *Tnfaip3*, *Tnfrsf14*, *Tnfrsf1a* and *Traf3a* are genes that are involved in the tumor necrosis factor signaling processes. TNF signaling is central to MS and EAE pathology. Thus it comes as no surprise that in the pEAE mouse model TNF was identified as the most significantly regulated upstream regulator in the IPA analysis (data not shown, available on request). The transcription factors *Hhex*, *Irf8*, *Maf*, *Stat3*, *Zfp36l1*, *Zfp36l2* and the transcription regulators *Ikzf1*, *Ptrf Sp110* were also upregulated in pEAE and although most of them are involved in immune-pathway transcription, their careful investigation could reveal additional functions. *Sox8*, the only transcription factor in the list which was downregulated in the pEAE mouse, promotes cellular differentiation and has a possible role in nervous system development.

MS susceptibility genes regulated in the pEAE without a clearly identified role in MS include the non-voltage gated sodium channel *Scnn1a*, the solute carrier *Slc44a2*, the lysosomal cysteine proteinase *Ctsh*, the serine biosynthesis enzyme *Phgdh*, and the mitochondrial *Hmgsc2*, an enzyme which controls ketogenesis. The proton sensing receptor *Gpr65*, involved in osteoclast differentiation, which is one of the most upregulated genes in the pEAE mouse model is also an MS susceptibility gene. *Iqgap1* is a scaffolding protein recognised as instrumental in cytoskeletal organisation and cell signaling [94]. *Rgs14*, a regulator of G protein signaling found upregulated in pEAE was identified as a gene differentially regulated in pathogenic CD4^+^ T cells [74]. Interestingly it is also recognised as a neuronal suppressor of long-term potentiation of synaptic transmission in the hippocampus [95]. The neuronal role of *Rgs14* opens a question as to whether its upregulation in pEAE may involve neuronal processes. Quantification of *Rgs14* levels in neuronal cells in MS or EAE will help to resolve this question. The same applies to *Scap2*, a gene with documented expression in both neurones and immune cells. *Scap2* is a Src kinase-associated phosphoprotein involved in integrin-stimulated cytoskeleton rearrangement [96], but it is also involved in neuronal differentiation [97]. A glioblastoma susceptibility locus, *Phldb1* [98] was also present in the list of MS susceptibility genes downregulated in the pEAE mouse. The identification of these MS susceptibility genes in the pEAE mouse model opens the door to their functional study in a mouse model which recapitulates some of the important clinical features of MS.

### Conclusion

The aim of this study was to transcriptionally characterise the progressive Biozzi ABH EAE mouse model in an unbiased manner and identify genes and disease processes that may be specific to this mouse model and that may recapitulate some of the MS features more adequately than other disease models. Importantly, genes specifically regulated in the chronic phenotype of EAE were identified and their role in the chronic disease phase as well as their potential as therapeutic targets can be studied further.

## Supporting Information

S1 Fig Heatmap of gene expression between samples. Heatmap of significantly regulated gene expression demonstrating the hierarchical clustering within the three control (1CTR - 3CTR) and three pEAE (1EAE– 3EAE) spinal cord samples (top dendrogram). The left hand side dendrogram demonstrates clustering between genes as predicted in the R arlgorithm. Scale: Yellow indicates high expression and red demonstrates low expression.
(TIFF)

S1 Table Significantly upregulated genes. (XLSX)

S2 Table Significantly downregulated genes. (XLSX)

S3 Table Comparison between gene expression in MOG EAE and pEAE. (XLSX)

S4 Table Comparison between gene expression in EAE and pEAE. (XLSX)

S5 Table Comparisons between pEAE pathways and Macrophage RNAseq pathways. (XLSX)

S6 Table All unique pEAE pathways after comparison with Macrophage RNAseq. (XLSX)
